# A2 MUCUS DEFICIENCY IMPACTS INTESTINAL EPITHELIAL CELL PROLIFERATION INDEPENDENT OF THE MICROBIOTA

**DOI:** 10.1093/jcag/gwab049.001

**Published:** 2022-02-21

**Authors:** X Han, K Tsai, J M Allaire, S M Crowley, A Lorentzian, C Maxwell, B Vallance

**Affiliations:** Pediatrics, University of British Columbia, Vancouver, BC, Canada

## Abstract

**Background:**

Intestinal homeostasis is highly dependent on the proliferation and differentiation of intestinal epithelial cells (IEC). IEC arise from intestinal stem cells (ISCs) that reside at the bottom of intestinal crypts. Following proliferation, the IEC migrate up as transient amplifying (TA) cells, and differentiate into mature IEC subtypes. When this process is disrupted, it can lead to aberrant IEC proliferation and differentiation. Mucus production by secretory goblet cells is also crucial for intestinal homeostasis, as mucus separates the IEC from luminal microbiota. Surprisingly, mice lacking Muc2, the main protein component of mucus, display increased distal colonic IEC proliferation and crypt hyperplasia at baseline, suggesting a relationship between Muc2 production and IEC proliferation.

**Aims:**

We investigated how mucus production impacts IEC proliferation and differentiation in the intestinal crypt.

**Methods:**

We used wildtype (Muc2^+/+^) and Muc2 deficient (Muc2^-/-^) littermates to measure distal colon crypt length and IEC proliferation pattern via microscopy. Organoids were also derived from distal colons of Muc2^+/+^ and Muc2^-/-^ mice, and quantified for size, density and proliferation for 7 days to test whether the hyper-proliferation phenotype was also seen in vitro, thus epithelial-intrinsic. Crypts and organoids were collected for RNA sequencing to examine changes in IEC proliferation pathways. Proliferation assessments were repeated in germ-free (GF) Muc2^+/+^ and Muc2^-/-^ mice. Muc2^-/-^ mice were also cross-bred with Lgr5^-EGFP-IRES-CreERT2^ mice to investigate the contribution of ISCs to IEC hyperproliferation resulting from Muc2 deficiency.

**Results:**

Significant crypt hyperplasia was observed in the distal colons of Muc2^-/-^ mice in concert with a > twofold increase in Ki67^+^ TA cells as compared to Muc2^+/+^ mice. Similarly, Muc2^-/-^ organoids also displayed significantly greater size, density and an increased number of Ki67^+^ cells than Muc2^+/+^ organoids. Hyperproliferation was also seen in GF mice and organoids, suggesting that mucus impacts IEC proliferation independent of the microbiome. Muc2^-/-^ Lgr5-EGFP^+^ mice showed no significant increase in numbers of Lgr5^+^ cells, indicating that Muc2 deficiency does not directly impact ISC number, but rather their proliferation and differentiation reflected in increased numbers of TA cells. RNA-sequencing results suggested that changes in lipid metabolism may underlie the increased IEC proliferation seen in Muc2^-/-^ mice.

**Conclusions:**

Mucus not only promotes IEC homeostasis by separating luminal bacteria from the intestinal epithelium, but also intrinsically modifies IEC proliferation independent of the microbiota. Taken together, our results emphasize the importance of mucus in controlling gut health through mechanisms independent of its role in barrier function.

**Funding Agencies:**

CCC, CIHRC.H.I.L.D Foundation

